# Supplementary material for: Theta-burst TMS to the posterior superior temporal sulcus decreases resting-state fMRI connectivity across the face processing network
Source: Netw Neurosci. 2020 Aug 1;4(3):746–60. doi: 10.1162/netn_a_00145 (PMC7462428; doi:10.1162/netn_a_00145)
Supplement: Supplementary file 1 [file netn-04-746-s001.pdf]

Handwerker, D. A., Ianni, G., Gutierrez, B., Roopchansingh, V., Gonzalez-Castillo, J., Chen, G., Bandettini, P. A., Ungerleider, L. & G., Pitcher, D. (2020). Supporting information for "Thetaburst TMS to the posterior superior temporal sulcus decreases resting-state fMRI connectivity across the face processing network." *Network Neuroscience*, *4*(3) 746-760. https://doi.org/10.1162/netn_a_00145

Supplemental Figure 1

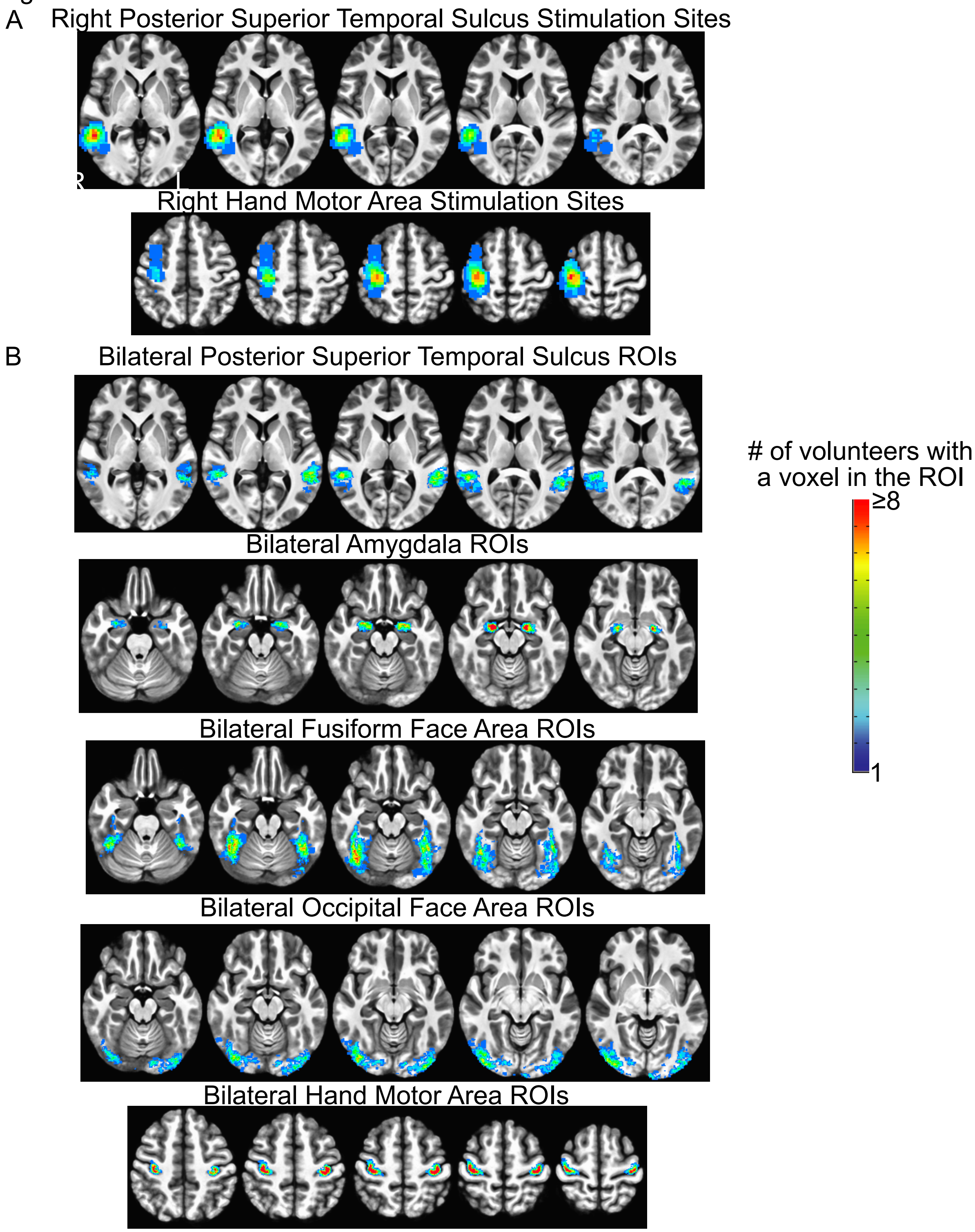

Union of the stimulation sites and ROI locations across volunteers. The anatomical underlay is the average of the aligned anatomical scans from the volunteers in this study. The overlay is the sum of all the volunter's ROIs. (A) The two stimulation sites across the subjects. The stimulation sites for each volunteer are visualized as a 1cm radius sphere centered on the stimulation site. The ROIs were individually localized for each stimulation site. The rpSTS stimulation site used a functional localizer and the motor stimulation site was defined using subject-specific anatomical landmarks. A re-examination of anatomical motor areas in each volunteer shows that the focus of the motor stimulation site was outside of the hand motor area for 5 volunteers with the focus being greater than 1 cm from the hand motor area in two volunteers. (B) Bilateral ROIs used for all connectivity analyses in this manuscript. All ROIs except for the hand motor areas were defined using the intersection of a Freesurfer-defined anatomical ROI and a Faces>Objects functional localizer

Supplemental Figure 2

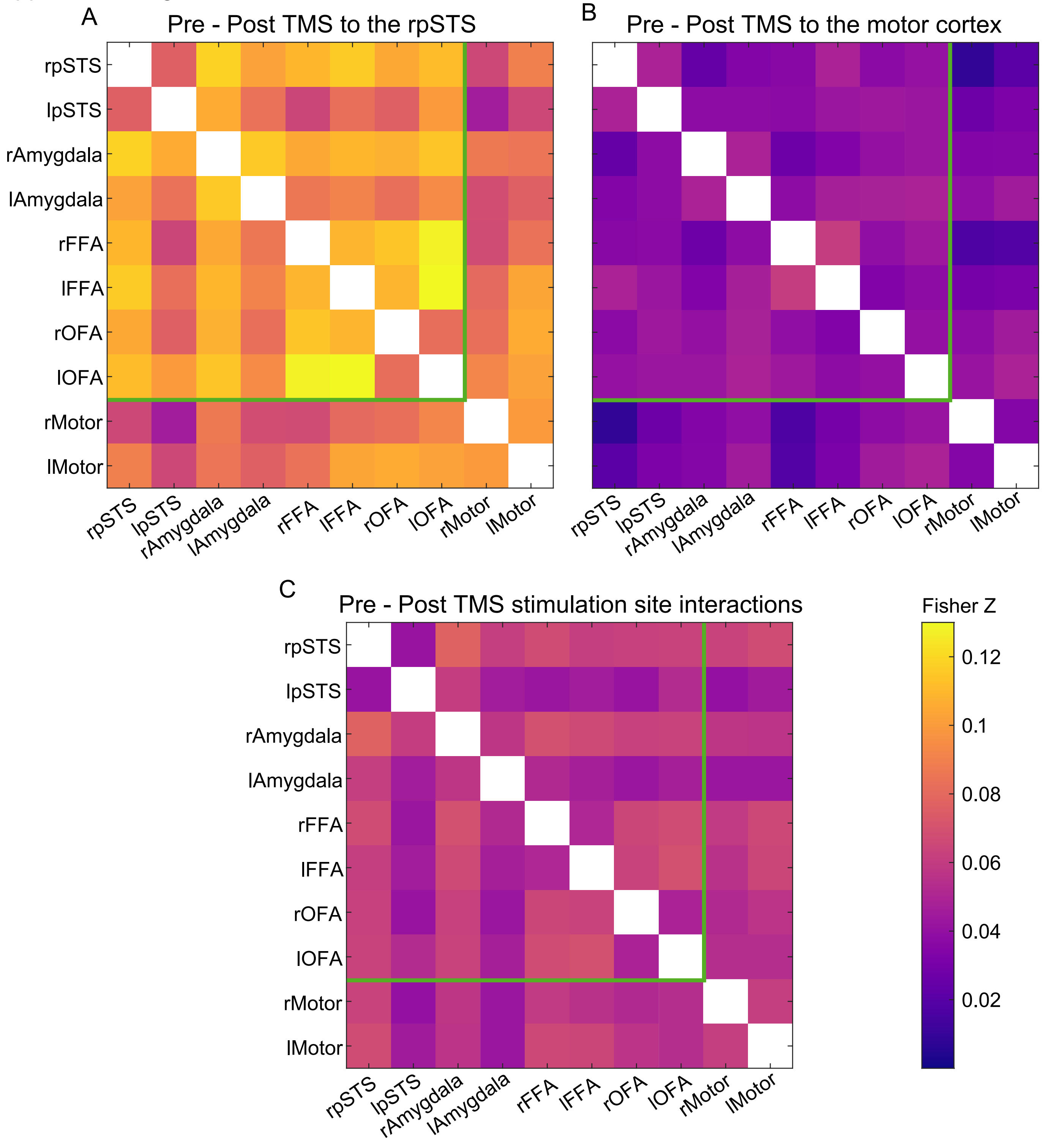

Fisher Z transformed correlation magnitude changes from pre - post TMS (A) To the rpSTS (B) To the the motor cortex, and (C) Pre - post stimulation & simulation site interaction effect. Magnitudes are the Matrix Based Analysis (MBA) model fits across the population. These are the results of the same analysis presented just for TMs to the rpSTS in Figure 1. Unlike Figure 1, the sizes of the squares are not scaled by their posterior probability. For the Motor cortex effect and the interaction effect, all posterior probabilities were less than 85%. The minimum value of the color scale is Z=0 rather than 0.2 in Figure 1 to better visualize the very small magnitude variations for motor stimulation and the interaction effects. The green line marks the ROI pairs that are within the pre-defined face-selective network.

Supplemental Figure 3

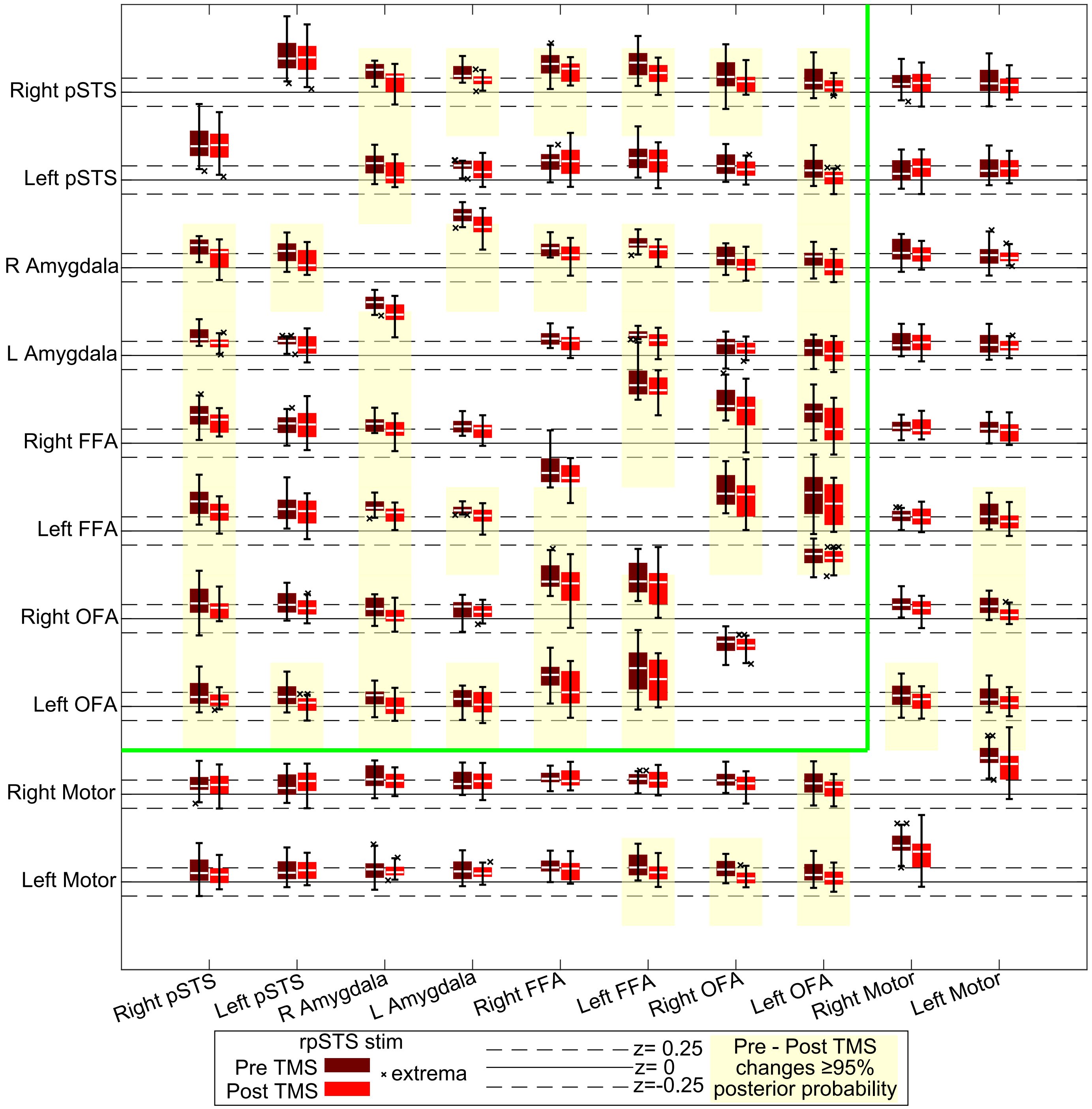

Correlations between the all pairs of ROIs in a pre-defined face network (within green line) as well as the bilateral primary motor hand regions. These boxplots correspond to the same data as the rpSTS stimulation box plots in Figure 2 but for the entire matrix of ROI pairs as in Figure 1. The first row directly corresponds to the rpSTS correlations shown in Figure 2. The solid and dashed black lines demarcate -0.25<z<0.25 for each row. Magnitudes are the MBA model fits across the population for each condition. Boxplots show 25-75% of the distribution. The white line is the median. Whiskers are the maximum and minimum values excluding outliers. MBA was used to calculate posterior probabilites that a difference is greater than 0. The ROI pairs with a yellow background show when the posterior probability was greater than 95% (Same as * in Figure 1)

Supplemental Figure 4

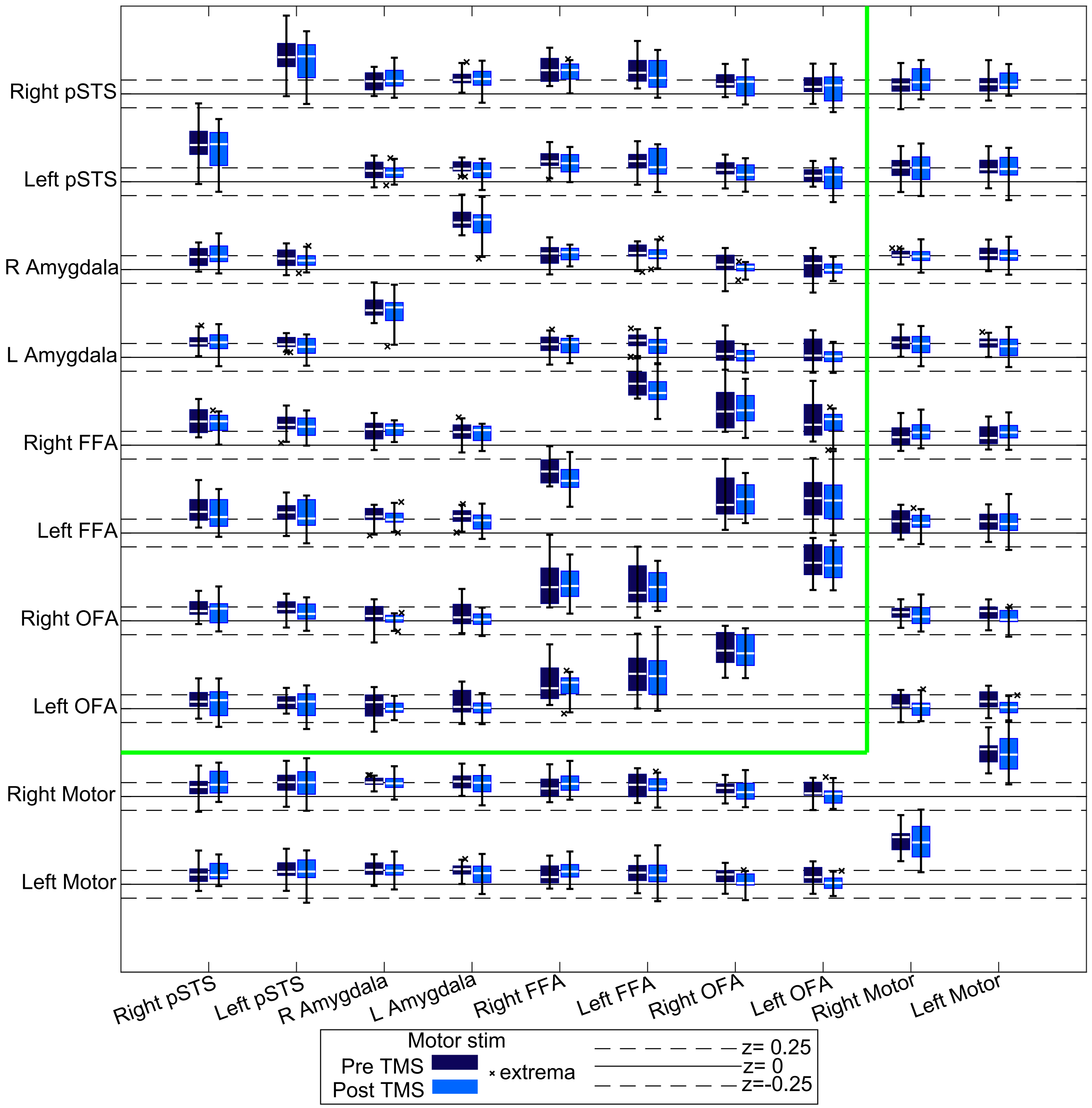

Correlations between the all pairs of ROIs in a pre-defined face network (within green line) as well as the bilateral primary motor hand regions. These boxplots correspond to the same data as the motor stimulation box plots in Figure 2 but for the entire matrix of ROI pairs as in Figure 1. The first row directly corresponds to the rpSTS correlations shown in Figure 2. The solid and dashed black lines demarcate -0.25<z<0.25 for each row. Magnitudes are the MBA model fits across the population for each condition. Boxplots show 25-75% of the distribution. The white line is the median. Whiskers are the maximum and minimum values excluding outliers. MBA was used to calculate posterior probabilites that a difference is greater than 0. None of the pre - post TMS to right motor cortex changes are above 85% posterior probability.
